# The Temporal Voice Areas are not “just” Speech Areas

**DOI:** 10.3389/fnins.2022.1075288

**Published:** 2023-01-04

**Authors:** Régis Trapeau, Etienne Thoret, Pascal Belin

**Affiliations:** ^1^La Timone Neuroscience Institute, CNRS and Aix-Marseille University, UMR 7289, Marseille, France; ^2^Aix-Marseille University, CNRS, UMR7061 PRISM, UMR7020 LIS, Marseille, France; ^3^Institute of Language, Communication and the Brain (ILCB), Marseille, France; ^4^Department of Psychology, Montreal University, Montreal, QC, Canada

**Keywords:** voice, speech, Temporal Voice Areas, functional MRI, humans, decoding, representational similarity analysis

## Abstract

The Temporal Voice Areas (TVAs) respond more strongly to speech sounds than to non-speech vocal sounds, but does this make them Temporal “Speech” Areas? We provide a perspective on this issue by combining univariate, multivariate, and representational similarity analyses of fMRI activations to a balanced set of speech and non-speech vocal sounds. We find that while speech sounds activate the TVAs more than non-speech vocal sounds, which is likely related to their larger temporal modulations in syllabic rate, they do not appear to activate additional areas nor are they segregated from the non-speech vocal sounds when their higher activation is controlled. It seems safe, then, to continue calling these regions the Temporal Voice Areas.

## 1. Introduction

It is a well-replicated finding that the Temporal Voice Areas (TVAs) of secondary auditory cortex are significantly more active in response to human voices compared to non-vocal environmental sounds (Belin et al., 2000; Kriegstein and Giraud, 2004; Andics et al., 2010; Frhholz and Grandjean, 2013; Pernet et al., 2015).

Neuroimaging voice localizers typically include speech in the human voice category of stimuli, as well as vocalizations with minimal linguistic content (here after, non-speech vocal sounds) such as coughs, laughs, or simple sustained vowels. TVA responses to non-speech vocal sounds are typically smaller than speech sounds (Belin et al., 2002; Fecteau et al., 2004; Bodin et al.'s, 2021), and in some cases not significantly stronger than control sounds (Belin et al., 2002). This has led some researchers to doubt that the TVAs are sensitive to vocal sounds, in general, and suggest that they are in fact Speech Areas, that is, responsive to the phonemic and/or semantic content of the input signal [e.g., component 5 in Norman-Haignere et al. (2015) study].

Yet, other results indicate that even non-speech vocal sounds induce greater TVA activity than control sounds (Bodin et al.'s, 2021) or lead to above chance classification into vocal/non-vocal categories (Rupp et al., 2022), suggesting a selectivity to this category of sounds in the TVAs.

Here, we provide a perspective on this issue by performing additional analyses of a published dataset (Bodin et al.'s, 2021), in which the same number (*n* = 12) of individual speech and non-speech vocal sounds were used along with 24 non-vocal sounds.

**Figure 1:**
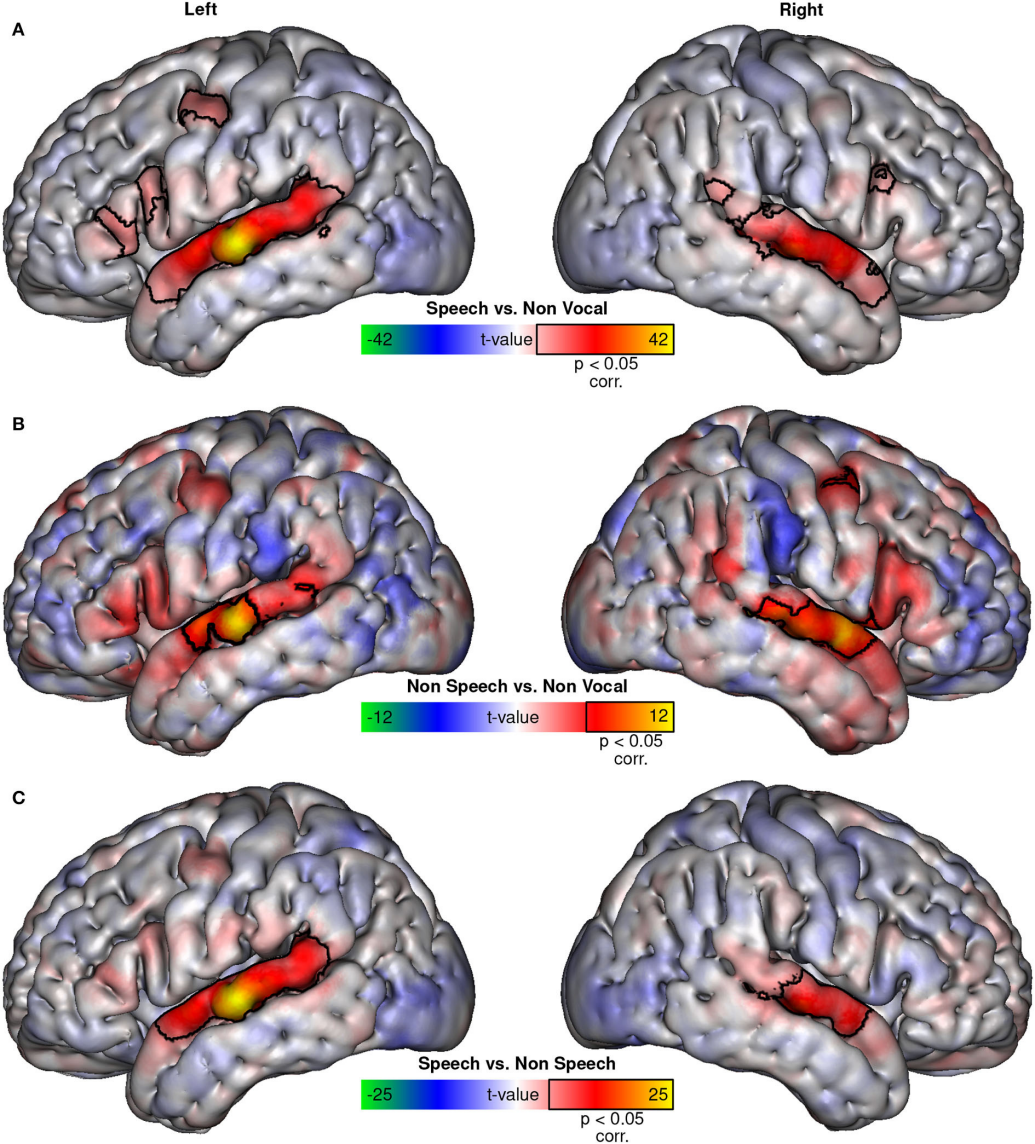
BOLD activations. Fixed-effects t-maps projected on the MNI152 surface of the speech vs. non-vocal contrast **(A)**, non-speech vs. non-vocal contrast **(B)**, and the speech vs. non-speech contrast **(C)**. Colormaps were adjusted to be symmetrical, with limits corresponding to the maximal *t*-value in each contrast. Areas with significant (*p* < 0.05, corrected) activation to each contrast are outlined in black.

Visualization using symmetrical colormaps (−max < *t*-value < max; allowing easy visual comparison of activation location differences between contrasts irrespective of significance threshold) of whole brain fixed-effects group t-maps of speech sounds vs. non-vocal sounds contrast (Figure 1A) and non-speech vocal sounds vs. non-vocal sounds contrast (Figure 1B) reveals topographically similar patterns of activation in both contrasts, suggesting that TVA activity is not limited to speech sounds. T-maps of both contrasts closely resemble those obtained by contrasting human voices vs. other types of sounds [compared with figure 1G from Bodin et al.'s (2021) study]. There is no clear visual evidence for supplementary regions recruited by speech stimuli, and both contrasts share the same maximum of activation in the left superior temporal gyrus. The main difference between the two contrasts is the higher general level of activation when using speech instead of non-speech vocal sounds. The speech vs. non-speech vocal stimuli contrast (Figure 1C) confirms this observation, as well as the apparent absence of additional regions activated by speech.

The larger general activation elicited by speech compared to non-speech vocal sounds might imply that speech sounds have a special status in the TVAs. To further investigate the role of speech and non-speech vocal sounds in the TVAs, we examined how a voice/non-voice decoder based on TVA activation performs for speech and non-speech vocal sounds, even when controlling for activation level differences between speech and non-speech. We also examined whether the representational geometry in the TVAs groups together speech and non-speech relative to non-vocal sounds.

## 2. Materials and methods

This analysis was performed on data collected in a previous study, which was designed for comparative neuroimaging between humans and nun-human primates (explaining the small sample size), but allowed distinct analyses of the activity evoked by speech and non-speech vocal sounds (Bodin et al.'s, 2021). Please refer to that study for a detailed description of materials and methods. The following sections present methodology that is specific to the present analysis.

### 2.1. Participants

Five native French human speakers were scanned [one man (author RT) and four women; 23–38 years of age]. Participants gave written informed consent and were paid for their participation.

### 2.2. Auditory stimuli

The analysis was performed on fMRI events corresponding to a subset of the stimulus set used in Bodin et al.'s (2021) study. Two main categories of sounds were used: human voices and non-vocal sounds, each containing 24 stimuli, for a total of 48 sound stimuli. Each main category was divided into two subcategories of 12 stimuli, forming four subcategories in total (cf. Supplementary Table 1). Human voices contained both speech [sentence segments from the set of stimuli used in Moerel et al.'s (2012) study, *n* = 12] and non-speech vocal sounds [vocal affect bursts selected from the Montreal Affective Voices dataset (Belin et al., 2008), *n* = 12].

Non-vocal sounds included both natural and artificial sounds from previous studies from our group (Belin et al., 2000; Capilla et al., 2013) or kindly provided by Petkov et al. (2008) and Moerel et al.'s (2012). Supplementary Figure 1 shows spectrograms and waveforms of the speech and non-speech vocal stimuli.

### 2.3. fMRI protocol

Detailed description of the fMRI protocol can be found in Bodin et al.'s (2021) study. In brief, functional scanning was done using an event-related paradigm with clustered-sparse acquisitions on a 3-Tesla MRI scanner (Prisma, Siemens Healthcare), equipped with a 64-channel matrix head-coil. To avoid interference between sound stimulation and scanner noise, the scanner stopped acquisitions such that three repetitions of a 500-ms stimulus (inter-stimulus interval of 250 ms) were played on a silent background. Then, seven whole-head functional volumes were acquired (TR = 0.945 s). Two functional runs, each containing one repetition of each stimulus, were acquired for each participant. Participants were instructed to stay still in the scanner while passively listening to the stimuli.

### 2.4. fMRI general linear modeling

General linear model estimates of responses to speech stimuli vs. non-vocal sounds, to non-speech vocal stimuli vs. non-vocal sounds, and to speech stimuli vs. non-speech vocal sounds were computed using fMRISTAT (Worsley et al., 2002).

### 2.5. Decoding

We tested whether support vector classification with a linear kernel [SVC: Chang and Lin (2011)] was able to predict, from beta values in primary auditory cortex (A1) and TVAs, whether fMRI events corresponded to the presentation of vocal or non-vocal sounds. We first tried this decoding using only speech vocal sounds and then using only non-speech vocal sounds. To have a balanced frequency in each category tested (*n* = 12), only half of the non-vocal sounds were used during classification. As the dataset consisted of sessions containing two functional runs during which a repetition of each stimulus was presented, we used a two-fold cross-validation, with each run serving successively as train and test sets. For each participant, the classifier was first trained on data from one functional run and tested on the other, and the other way around in a second fold. The reported classification accuracy is the average of the scores obtained in two-fold cross-validation. Above significance threshold in classification accuracy was determined by building a bootstrapped distribution of classification scores obtained on 100,000 iterations of two-fold dummy classification tests with random labels. Comparisons between different classification results were tested using Wilcoxon signed-rank tests.

### 2.6. Representational similarity analysis

Representations of dissimilarities within the stimulus set in A1 and TVAs were assessed using the representational similarity analysis (RSA) framework (Kriegeskorte et al., 2008; Nili et al., 2014). Representational dissimilarity matrices (RDMs) capturing the pattern of dissimilarities in fMRI responses, and generated by computing the Euclidean distance between stimuli in multi-voxel activity space, were compared with three binary categorical models: (1) a “human” model in which human voices are categorized separately from non-vocal sounds, with an equal contribution of speech and non-speech vocal stimuli; (2) a “speech” model categorizing speech apart from all other sounds (i.e., non-vocal and non-speech vocal stimuli); and (3) a “non-speech” model categorizing non-speech human voices apart from other sounds (i.e., non-vocal and speech stimuli).

We also compared brain RDMs with an acoustical RDM reflecting the pattern of differences between the modulation power spectra [Thoret et al. (2016); MPS: quantifies amplitude and frequency modulations present in a sound] of the 48 stimuli (see Supplementary Figure 2).

Planned comparisons were performed using two-sample bootstrapped *t*-tests (100,000 iterations, one-tailed) that compared the within vs. between portions of the brain and acoustical RDMs, as shown in Supplementary Figure 3.

### 2.7. Regions of interest

RSA and SVC were performed in two regions of interest (ROI): primary auditory cortex (A1) and Temporal Voice Areas (TVAs) in each hemisphere.

In each participant and hemisphere, the center of the A1 ROI was defined as the maximum value of the probabilistic map (non-linearly registered to each participant functional space) of Heschl's gyri provided with the MNI152 template (Penhune et al., 1996). The 57 voxels in the functional space that were the closest to this point and above 50% in the probabilistic maps constituted the A1 ROI.

In each participant and hemisphere, the TVAs' ROI was the conjunction of three TVAs (posterior, middle, and anterior). TVA locations vary from one individual to another and were therefore located functionally. The center of each TVA region corresponded to the local maximum of the *human voice* > *all other sounds* t-map [computed using both speech and non-speech events, see Bodin et al.'s (2021)], whose coordinates were the closest to the corresponding TVA reported in the study of Aglieri et al. (2018). The 19 voxels in the functional space that were the closest to this point and above significance threshold in *human voice* > *all other sounds* t-map constituted a TVA ROI. The TVAs' ROI for one hemisphere was the conjunction of the three TVA ROIs of 19 voxels, forming a ROI of 57 voxels.

### 2.8. Standardization

To assess the contribution of either categorical or topographical differences in stimulus activation, activity patterns of each ROI (*RSA*: 48 stimuli × 57 voxels; *SVC*: 96 events × 57 voxels) were standardized using two methods before running RSA and SVC: a standardization *along stimuli*, where *z*-scores were computed for each voxel along the stimulus (RSA) or event (SVC) dimension [which is the default standardization in machine learning packages; Pedregosa et al. (2011)], and a standardization *along voxels*, where *z*-scores were computed for each stimulus (or event) along the voxel dimension (see Supplementary Figure 4). For RSA, standardization was performed on activity patterns before computing RDMs. For SVC, standardization was performed on all events (both runs) before splitting data in train-test sets.

## 3. Results

### 3.1. Decoding stimulus categories

Decoding results are shown in Supplementary Figure 5. For both standardization methods, when attempting to classify fMRI events in speech or non-vocal categories, the SVC performed poorly in A1 and well above significance level in TVAs (mean scores for standardization method along stimuli and along voxels, respectively. A1: $\bar{x}=0.58$ and 0.57; TVAs: $\bar{x}=0.89$ and 0.84). When using non-speech events instead of speech events, performance in A1 remained poor and performance in TVAs dropped to values close to significance level (A1: $\bar{x}=0.56$ and 0.61; TVAs: $\bar{x}=0.65$ and 0.64). The differences in SVC performance when using speech or non-speech vocal stimuli were not significant for both A1 and TVAs. However, in the TVAs, classification accuracy was higher for speech than for non-speech vocal sounds for all the participants, suggesting that this difference may become significant with a larger sample of participants. The differences in SVC performance between standardization methods were not significant for both A1 and TVAs.

### 3.2. Representational similarity analysis

**Figure 2:**
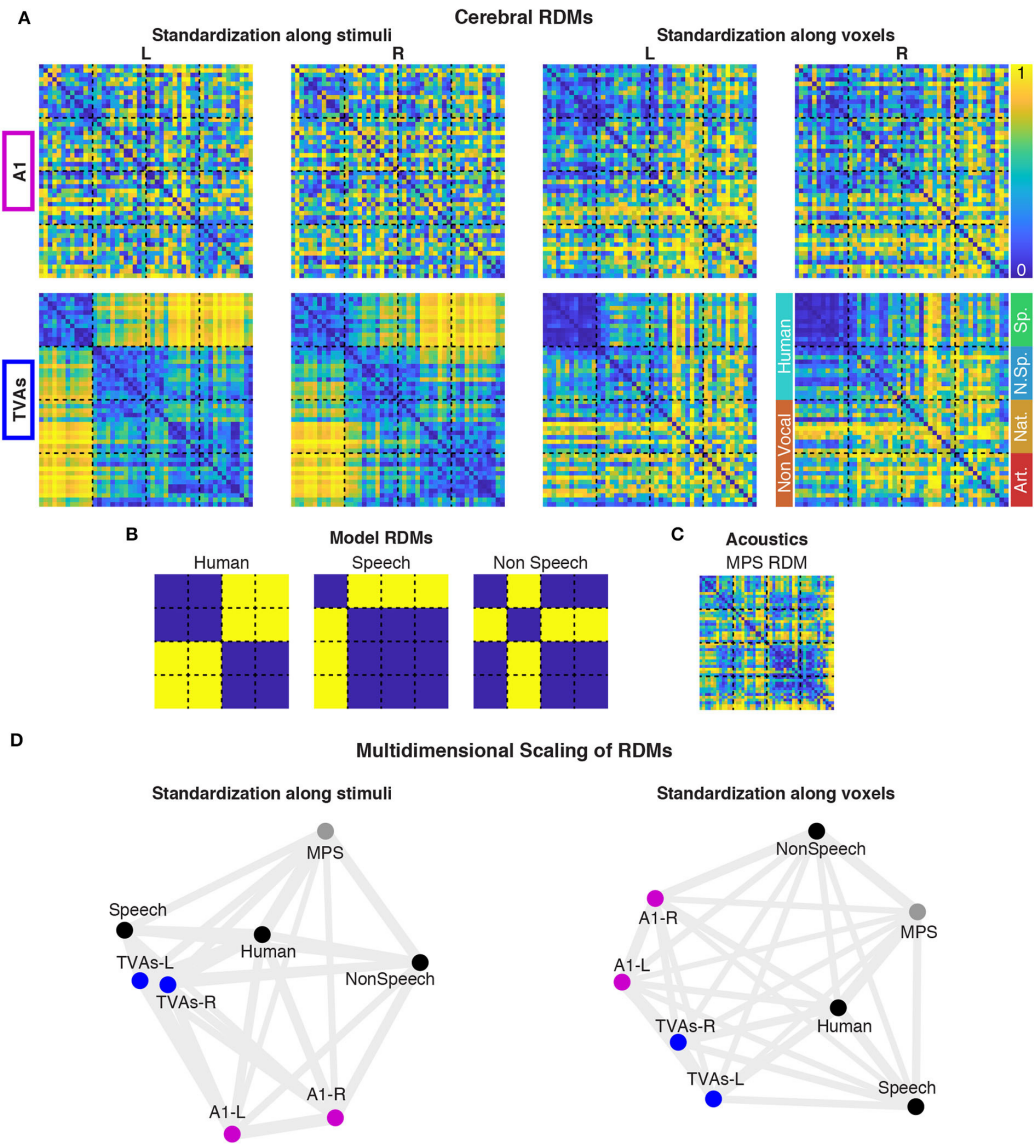
Representational similarity analysis (RSA) in A1 and the TVAs. Cerebral RDMs showing percentile dissimilarities in pairwise fMRI response to the 48 stimuli, for both ROIs and standardization methods **(A)**. Portions of the RDMs corresponding to the main and sub-categories of stimuli are indicated next to the bottom right RDM. Cerebral RDMs were compared (Spearman correlations) with three categorical model RDMs **(B)** and one acoustical RDM **(C)**, for each standardization method. These comparisons are represented *via* multidimensional scaling **(D)**.

The visual representation of the pattern of Spearman correlations among brain RDMs (Figure 2A), categorical models (Figure 2B), and acoustical RDM (Figure 2C) was performed *via* multidimensional scaling (MDS, Figure 2D) for both standardization methods.

Using standardization along stimuli, cerebral RDMs computed in the left and right TVAs cluster together close to the “speech” (especially for TVAs-L) and “human” categorical models, and separated from the “non-speech” categorical model, the acoustical model, or the A1 brain RDMs. All three planned comparisons (see Supplementary Figure 3) were significant in the TVA RDMs (all *p*-values are below 0.01 after Bonferroni correction for 24 comparisons), while nothing was significant in A1.

Using standardization along voxels, TVA RDMs are less separated from A1 RDMs and closer to the “human” than the “speech” model. Only speech vs. non-speech test was significant in the TVAs, while nothing was significant in A1.

## 4. Perspective

The univariate analysis suggests that speech sounds activate the same set of regions as non-speech vocal sounds, simply more strongly. There is no clear evidence of additional areas activated specifically by speech sounds, as shown in Figure 1C, in which the contrasts of speech vs. non-speech vocal sounds show the same distribution of regions as the classical speech vs. non-vocal sounds contrast. This voice network appears to be recruited by both speech and non-speech vocal sounds, but more strongly by speech sounds.

The classification analysis confirms this notion: while classification accuracy for vocal vs. non-vocal sounds was larger on average for speech than for non-speech vocal sounds, the difference was not significant (likely due, though, to our small number of participants), and both were above chance level. Controlling for differences in activation level between stimuli with the standardization along voxels did not change this pattern (Supplementary Figure 5).

The Representational Similarity Analysis helped refine this picture. While A1 RDMs did not show any similarity with any of the categorical model RDMs (Figure 2B), the TVA RDMs were strongly associated, in both hemispheres, with the “speech” model, categorizing speech apart from all other sounds including non-speech voice. However, when controlling for stimulus activation levels *via* the voxelwize standardization (Supplementary Figure 4), the picture changed and the “human” model, grouping speech and non-speech vocal sounds together and apart from the non-vocal sounds, was the most closely associated to both left and right TVAs.

Overall, our analyses indicate that speech does not have a special status compared to non-speech vocal sounds in the TVAs, apart from the fact that they drive them to a higher activation level. This particular result needs to be further investigated in future studies, but is likely related to the more complex spectro-temporal structure of speech compared to non-speech vocal sounds (Supplementary Figure 1), with more pronounced temporal modulations around 4 Hz, close to the syllabic rate in English, (Supplementary Figure 2). Spectro-temporal complexity is indeed known to increase the strength of activation in non-primary auditory fields (Samson et al., 2011). It seems safe, then, to continue calling these regions the Temporal Voice Areas. Furthermore, using the more encompassing term of “voice” instead of “speech” to name these areas, opens up more questions and hypotheses for future studies using dedicated experimental designs with larger sample size, that will help to understand how spectro-temporal complexity, linguistic content, or attention to distinct voice features (von Kriegstein et al., 2003) modulate the cortical processing of voice.

## Data availability statement

Publicly available datasets were analyzed in this study. This data can be found at: Zenodo: https://doi.org/10.5281/zenodo.5071389.

## Ethics statement

The studies involving human participants were reviewed and approved by Ethical board of Institut de Neurosciences de la Timone. The participants provided their written informed consent to participate in this study.

## Author contributions

PB and RT contributed to the conception and design of the study. RT and ET performed the statistical analysis. RT wrote the first draft of the manuscript. PB and ET wrote sections of the manuscript. All authors contributed to manuscript revision, read, and approved the submitted version.
