# Supplementary material for: The Temporal Voice Areas are not “just” Speech Areas
Source: Front Neurosci. 2023 Jan 4;16:1075288. doi: 10.3389/fnins.2022.1075288 (PMC9846853; doi:10.3389/fnins.2022.1075288)
Supplement: Supplementary file 1 [file Presentation_1.PDF]

Supplementary Material

1 SUPPLEMENTARY TABLES AND FIGURES

1.1 Tables

Table S1. Auditory stimuli, categories and subcategories

**Main category**		**Subcategory**	
**Name**	**#**	**Name**	**#**
Human	24	Speech	12
		Non Speech	12
Non Vocal	24	Natural	12
		Artificial	12

1.2 Figures

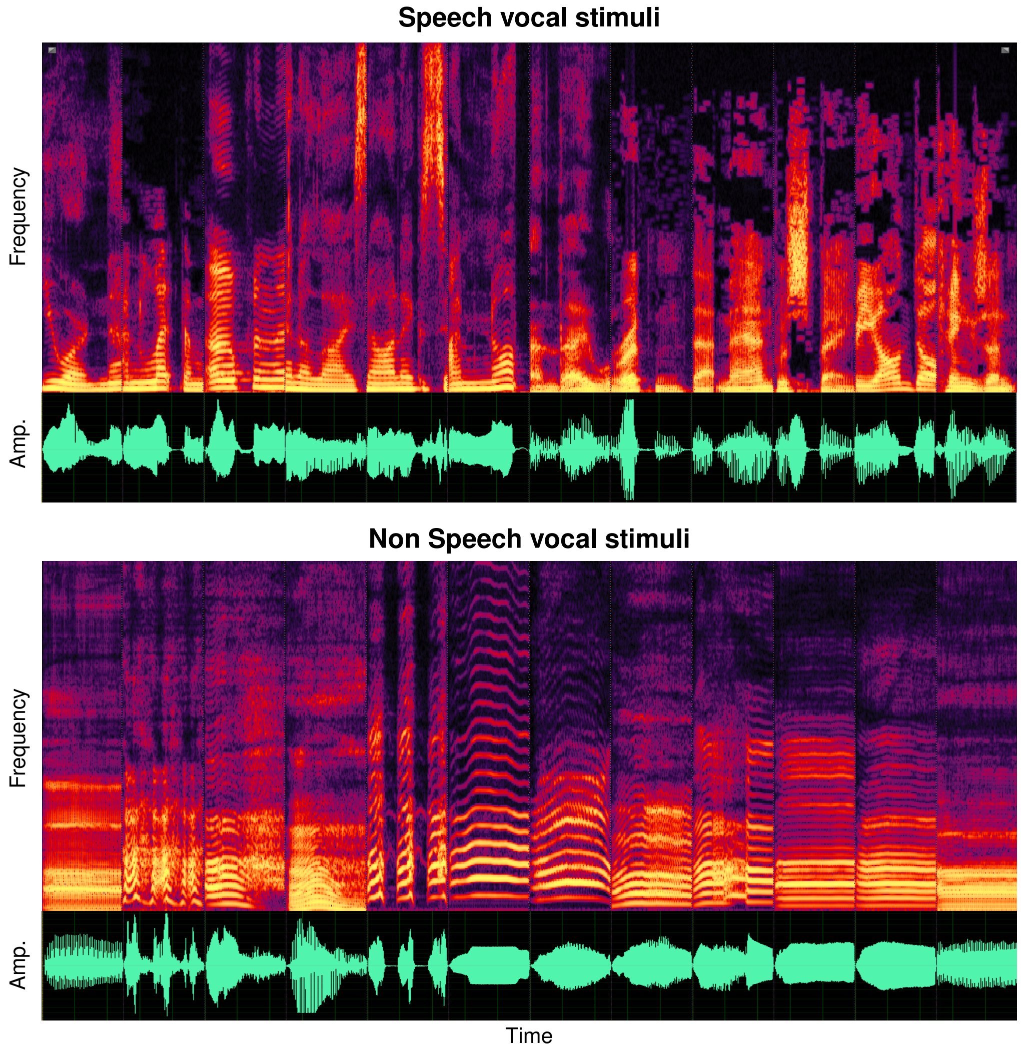

Figure S1. Spectrograms and waveforms of the speech and non-speech vocal stimuli. In each subcategory, all 12 stimuli are displayed one after the other.

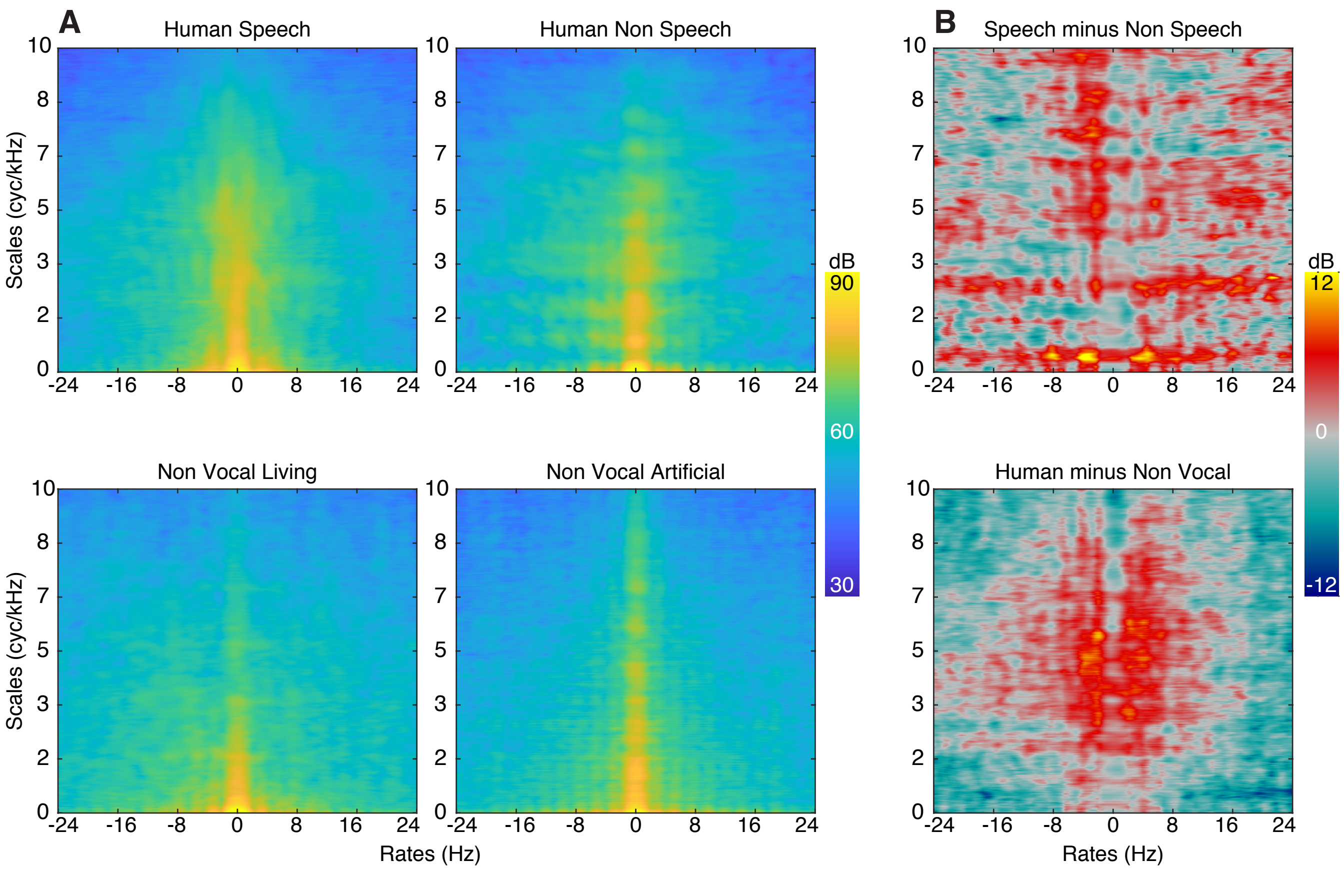

Figure S2. Modulation power spectra. Modulation power spectra of the four subcategories of the stimulus set (**A**). Difference between the MPS of the speech and non-speech vocal sounds (**B**, top panel). Difference between the MPS of human voices and non-vocal sounds (**B**, bottom panel)

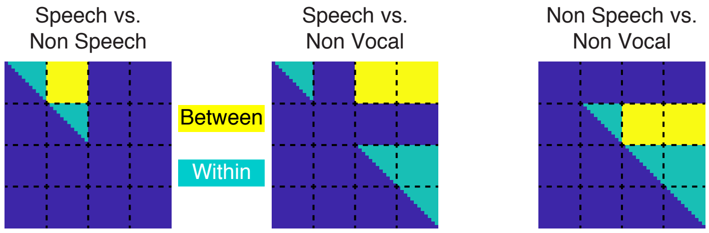

Figure S3. Between vs. within tests performed on the RDMs. We tested whether values inside the portions of the RDMs that corresponded to the dissimilarities *between* the categories planned to compare (e.g. speech vs. non-speech) were significantly different from values inside the portions of the RDMs that corresponded to the dissimilarities *within* these categories. ”Between” portions are in yellow, ”within” portions are in cyan.

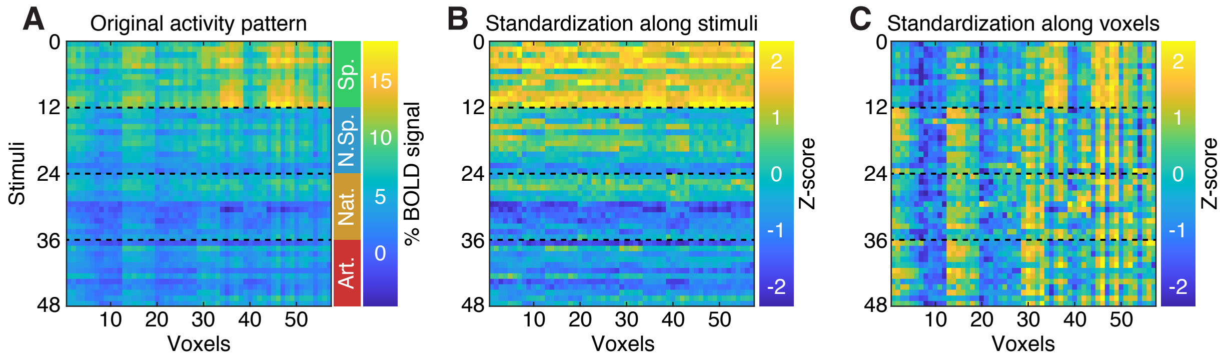

Figure S4. Standardization methods. Original activity pattern ($stimuli \times voxels$) in the left TVAs of the first participant (**A**). Same activity pattern standardized along the stimulus dimension (**B**) or along the voxel dimension (**C**).

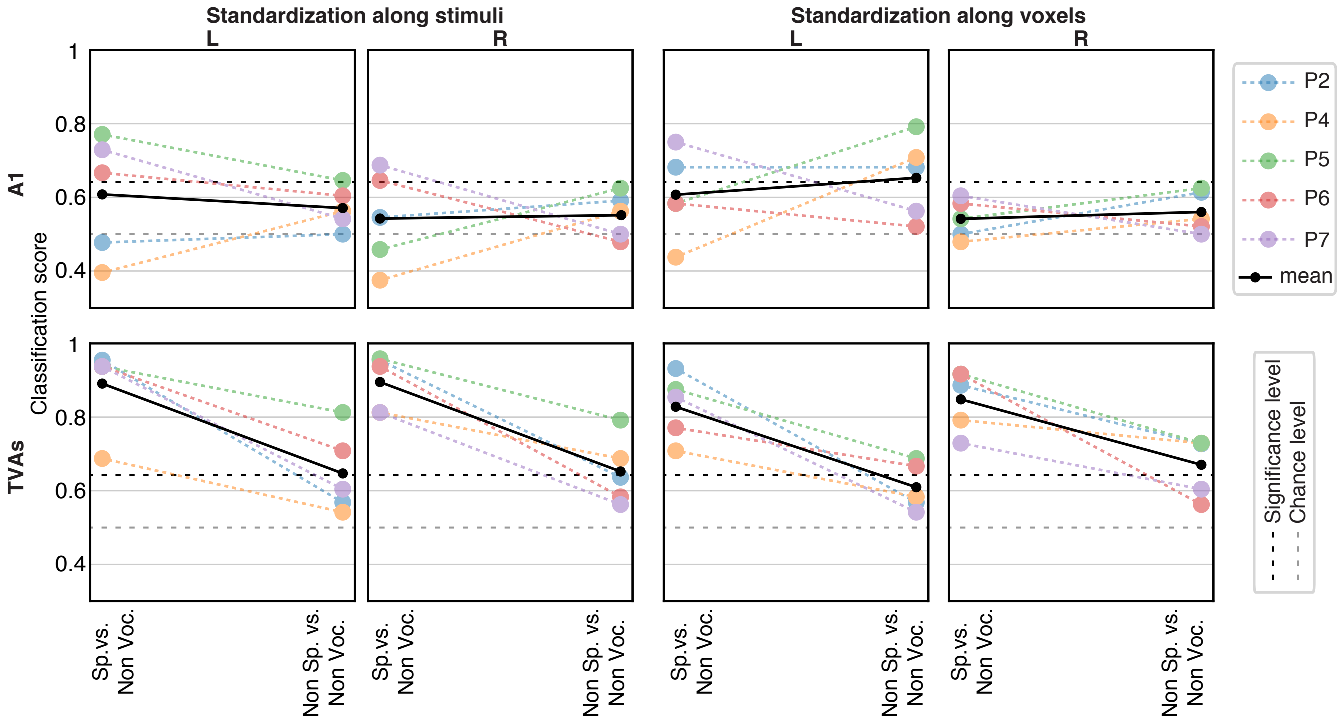

Figure S5. Support Vector Classification results. Each colored dot corresponds to the mean of a two-folds cross-validated classification for one participant, one ROI and one standardization method. Black dots are the means across participants. The horizontal gray and black dashed lines indicate chance (0.5) and significance (0.642) levels, respectively.
